# Harnessing intra-varietal variation for agro-morphological and nutritional traits in a popular rice landrace for sustainable food security in tropical islands

**DOI:** 10.3389/fnut.2023.1088208

**Published:** 2023-02-23

**Authors:** Raj Kumar Gautam, Pankaj Kumar Singh, Kannan Venkatesan, Bandol Rakesh, Krishnan Sakthivel, Sachidananda Swain, Muthulingam Srikumar, S. K. Zamir Ahmed, Kishnamoorthy Devakumar, Shyam Sunder Rao, Joshitha Vijayan, Sharik Ali, Sapna Langyan

**Affiliations:** ^1^ICAR-Central Island Agricultural Research Institute, Port Blair, Andaman and Nicobar Islands, India; ^2^ICAR-National Bureau of Plant Genetic Resources, Pusa, New Delhi, India; ^3^ICAR-Indian Institute of Oilseed Research, Hyderabad, Telangana, India; ^4^ICAR-Sugarcane Breeding Institute, Coimbatore, Tamil Nadu, India; ^5^ICAR-National Institute of Plant Biotechnology, Pusa, New Delhi, India

**Keywords:** rice landraces, open florets, biochemical traits, intra-varietal variations, sustainable food system

## Abstract

**Introduction:**

Rice crop meets the calorie and nutritional requirements of a larger segment of the global population. Here, we report the occurrence of intra-varietal variation in a popular rice landrace C14-8 traditionally grown under the geographical isolation of the Andaman Islands.

**Methods:**

Based on grain husk color, four groups were formed, wherein the extent of intra-varietal variation was studied by employing 22 agro-morphological and biochemical traits.

**Results:**

Among the traits studied, flavonoid and anthocyanin contents and grain yield exhibited a wider spectrum of variability due to more coefficients of variation (>25%). The first five principal components (PCs) of principal components analysis explained a significant proportion of the variation (91%) and the first two PCs explained 63.3% of the total variation, with PC1 and PC2 explaining 35.44 and 27.91%, respectively. A total of 50 highly variable SSR (HvSSR) markers spanning over 12 chromosomes produced 314 alleles, which ranged from 1 to 15 alleles per marker, with an average of 6.28. Of the 314 alleles, 64 alleles were found to be rare among the C14-8 selections. While 62% of HvSSR markers exhibited polymorphism among the C14-8 population, chromosomes 2, 7, 9, and 11 harbored the most polymorphic loci. The group clustering of the selections through HvSSR markers conformed to the grouping based on grain husk coloration.

**Discussion:**

Our studies on the existence and pertinence of intra-varietal variations are expected to be of significance in the realms of evolutionary biology and sustainable food and nutritional security under the changing climate.

## 1. Introduction

Although the rice crop contributes immensely to the world’s food supply, especially in Asia, its history of origin, domestication, and evolutionary genetics remain elusive (1, 2). It is believed that rice was domesticated approximately 10,000 years ago from its wild ancestor, *Oryza rufipogon*, a widely distributed Asian native species (3, 4). Rice landraces constitute an important gene pool for genetic improvement for the present as well as future needs (5). However, the genetic diversity of domesticated rice has been reduced to the tune of 80% compared with wild progenitor right from domestication to the development of modern cultivars (6, 7). If the present trend continues, it may deprive mankind of the genetic advantage of rice diversity in meeting the unforeseen challenges of diseases, pests, agronomic adaptation, and climatic change (8). Traditional farmers in different parts of the world prefer landraces due to their time-tested adaptations to local climates and specific traits (9, 10), and therefore, detailed profiling of these landraces with respect to agro-morphological, biochemical, and genomic characterization is very essential (11, 12). Singh (13) opined that the genetic diversity of the heterogenous population confers resilience and adaptive traits to the species.

Traditional landraces having distinct morphological traits and locally popular names based on their novelties and representing the intermediate stage between a wild ancestor and modern cultivars serve as reservoirs of various useful genes (14). Although landraces have a lower yielding ability, these play an important role in maintaining yields in traditional and stress-prone agricultural systems (15, 16). Such ancient varieties have evolved under a series of climatic, management, and cultural events over the long period of domestication, developing genetic resilience to climatic upheavals (17). Kyratzis et al. (18) opined that the intra-varietal variation found within landraces offers the scope of genetic plasticity, and thus, landraces have the ability to adapt to local field conditions and marginal environments. Numerous studies confirm that locally adapted crops and their landraces are time-tested and deeply seated in the local livelihood system, which requires low inputs, provides better nutrition, and is resistant to prevailing abiotic and biotic stresses, particularly in marginal areas (16, 19). Therefore, it is worthwhile to improve farmers’ genetic resources not only for indigenous requirements but also for trait improvement of other ecosystems (9, 20). However, it is significant to mention that all the landraces, no matter how useful these could be in future, may be expected to be conserved under on-farm conditions by farmers themselves even if these are low yielding in heterogeneous form, and seed purity and availability are the concerning issues (21).

It is recently reported that natural evolution and artificial selection may cause speciation in rice (22). While traditional landraces have been reconstituted through the interplay between adaptation to the local environments and selection exercised by farmers as per their agro-ecological and cultural requirements and preferences, wild rice populations proliferate owing to their invasive attributes, and outcompeting ability under natural conditions (23). However, it is not understood clearly how these events interacted to shape and influence the population genetics and intra-varietal structure of a landrace over time and space (18, 24). Although landraces constitute genetically a dynamic system, there are scanty reports on the systematic characterization and quantification of such intra-varietal diversity in their hot spot areas (10). Their detailed characterization and exposition for a series of traits and selection of desirable types may be vital for understanding evolutionary trajectory, trait discovery, and crop improvement (25).

**Figure 1:**
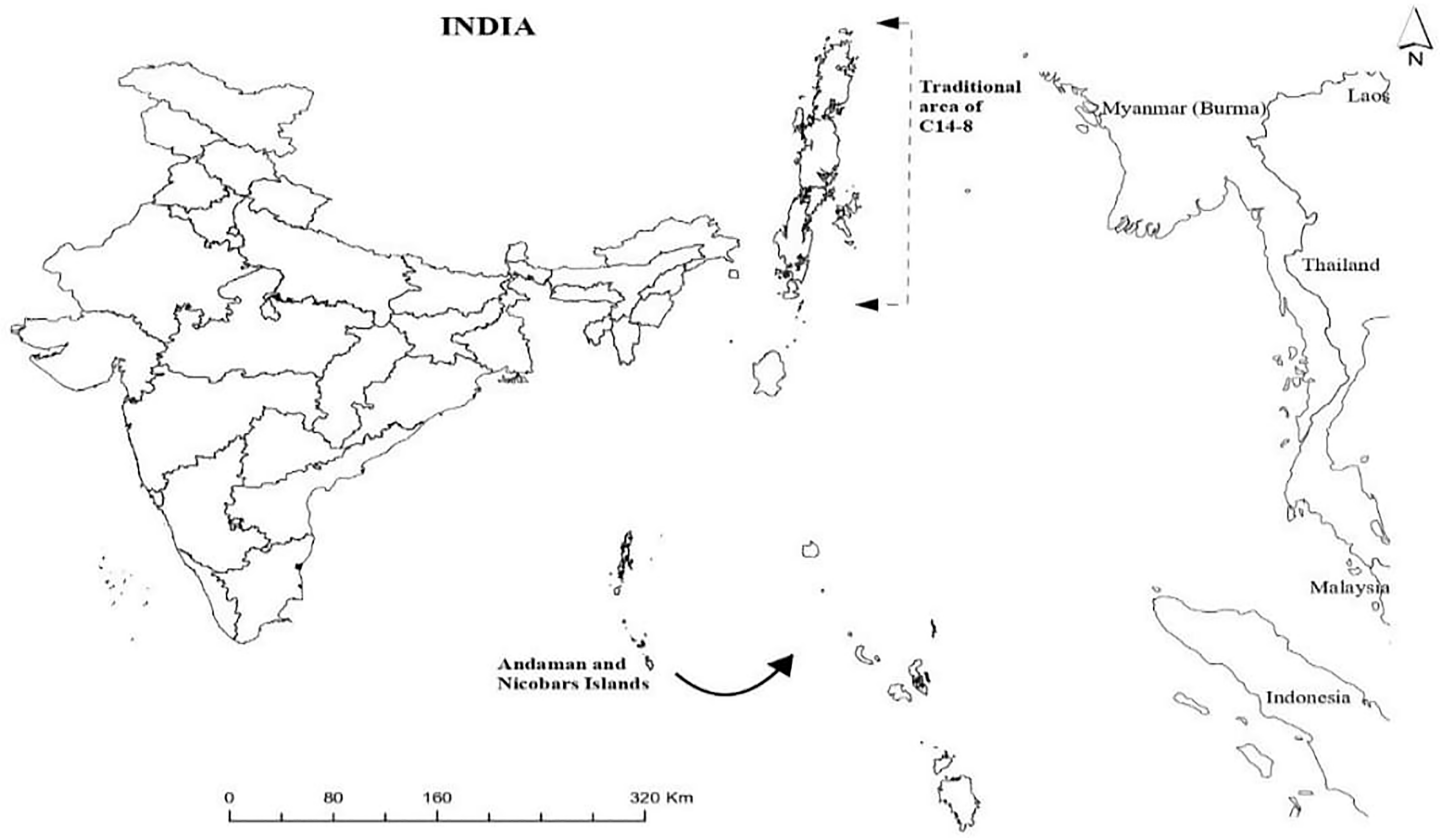
Map showing Andaman and Nicobar Islands.

A traditionally popular C14-8 rice landrace has carved out a historical niche in the agricultural landscape of the tropical Andaman and Nicobar Islands due to its advantageously adaptive traits albeit its precise origin and introduction in the islands are not clearly known (26). Londo et al. (7) and Lu (27) concluded that the domestication of rice occurred at least twice, once in the south of Northeastern India, Myanmar, and Thailand and again in Southern China. Here, it is pertinent to mention that the Andaman Islands where C14-8 has been traditionally grown since 1945 or before are also geographically located within the aforementioned first center of domestication [(28); Figure 1]. The tropical climate of the islands is also understood to favor higher diversification rate during the evolutionary process due to biological process of reproductive isolation, faster genetic evolution, and dynamic abiotic and biotic pressures (28, 29).

**Figure 2:**
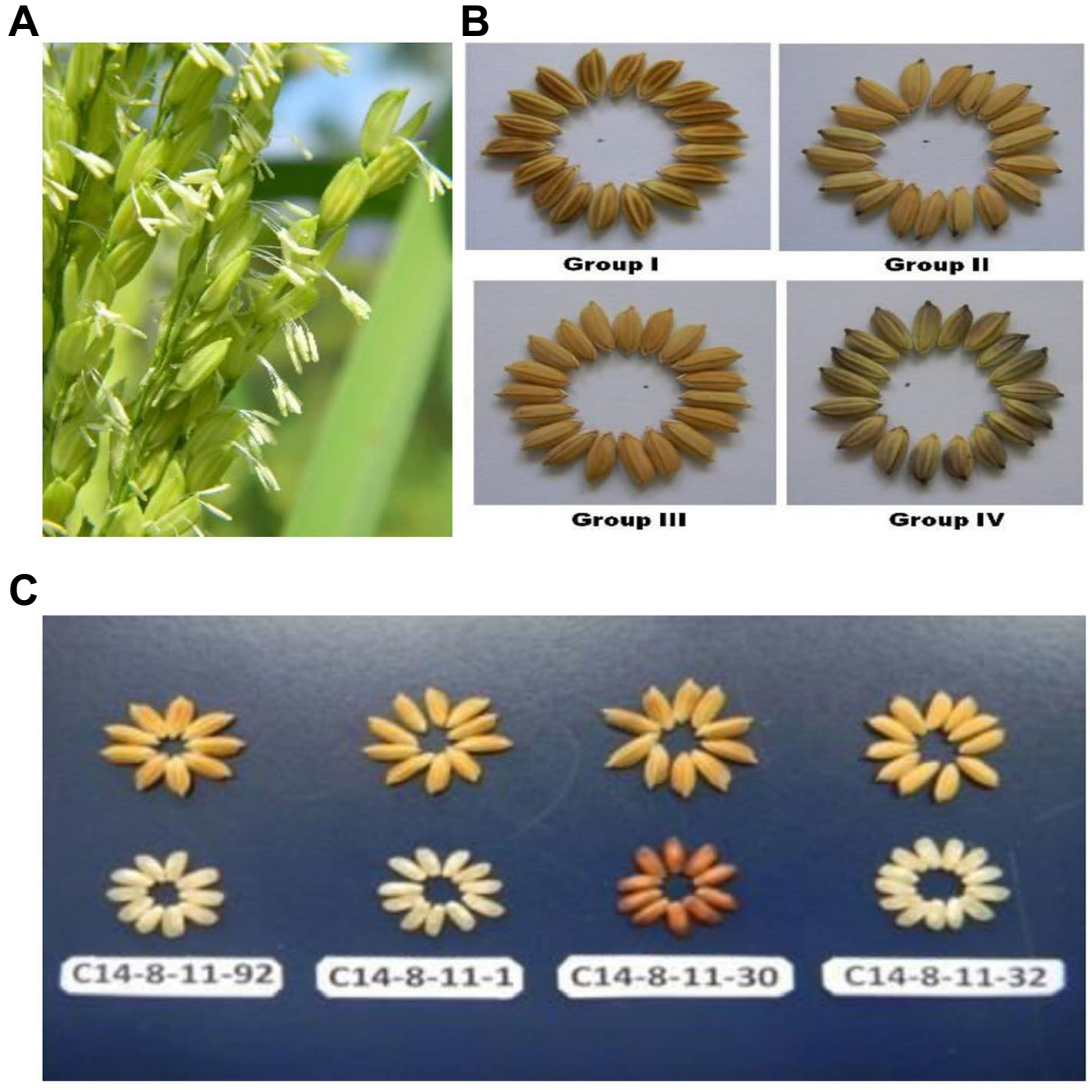
**(A)** Rice florets expressing wider opening with extra-long filaments and protruding anthers. **(B)** Rice grains having different husk color in Group I (brown husk with white apiculus), Group II (yellow husk with black apiculus), Group III (yellow husk with Yellow apiculus) and Group IV (Golden furrows on brown husk with Golden furrows on brown husk with black apiculus). **(C)** Classification of C14-8 selections into 4 types of colors in husk and corresponding decorticated rice grains.

Although rice is a cleistogamous crop with almost complete autogamy, a peculiar tendency for an open floret was observed in C14-8 (Figure 2A). The genetic and molecular bases of an open floret trait in rice have been unraveled and mostly attributed to jasmonic acid pathways manipulations (30). Furthermore, the detection of different grain color types (Figures 2B,C) occurring naturally within promiscuous C14-8 landrace population prompted us to further investigate the causes, nature, and extent of genetic diversity prevailing in a traditional rice landrace grown under geographical and phenological isolation through the use of morphological, biochemical, and molecular markers. Furthermore, it was intriguing to know if the classification for grain husk color conformed to the cluster grouping based on the agro-morphological, biochemical, and molecular markers. Our study also revealed the traits, which underwent relatively wider divergence as a result of natural selection and adaptation under low input agronomic management for over 7 decades under geographical isolation of islands. It is also imperative to understand the dynamics of inter-trait correlation coefficients in such a biologically unique population for their utilization during indirect selection.

Although there is a good number of studies on the diversity between varieties, pure lines, local cultivars, and/or landraces [(31–38); Vannirajan et al., 2012], systematic studies on intra-varietal variation in landraces are far more inadequate. Therefore, the novelty of our study is the documented multi-trait attempt to understand the promiscuity-induced intra-varietal genetic diversity for 22 agro-morphological, biochemical, and molecular traits in an open floret tropical *japonica* rice variety being cultivated over 7 decades under the closed system of tropical islands. The model on understanding the biological basis and evolutionary genetics of these findings could be relevant and extrapolated to other self-pollinated crops with similar traits and conditions as well. It is also important to know which traits or combinations of traits contributed to maximum variation in a landrace population. The information, thus, generated could be utilized for pinpointing responsible traits useful in direct selection for genetic improvement of such a population. In addition, it is also interesting to look for promising lines in the germplasm pool that possess superiority for the maximum number of agronomic and biochemical traits. The incremental gain in the production potential of socially popular C14-8 rice landrace through our selection interventions will be realized without additional cost to the farmers and with no adverse impact on the local environment and the prevailing food system. In addition to their use for a basic understanding of evolutionary genetic perspectives and mapping studies, potential genotypes emanating from such landraces are strongly likely expected in realizing the Sustainable Development Goals (SDG) by 2030.

## 2. Methodology

### 2.1. About the study locale

The tropical Andaman and Nicobar Islands encompassing more than 500 islands located in the Bay of Bengal are approximately 1,200 km far from mainland India (Figure 1). In view of pristine oceanic and terrestrial life forms, Andaman and Nicobar Islands are the first in India to have been listed as the new “hope spots” by the International Union for Conservation of Nature.^1^ The Andaman and Nicobar archipelago is positioned between two major biodiversity areas of long Island Arch extending from the Arakan Yoma hill region of Myanmar to the Sumatran range of Indonesia (39) and rich in tropical plant diversity representing Indian, Burmese, Thai, Malaysian, and Indonesian floras (39). These islands are also listed as one of the 22 agrobiodiversity hot spots in India.

### 2.2. Salient features of C14-8 landrace

C14-8, locally called *Aath Number Dhan* is a highly popular rice landrace owing to its agronomic merits and ability to fit in the geo-climatic and socioeconomic conditions of the Bay Islands. It is suitable for high rainfall conditions (3,000 mm annual rainfall) of tropical islands and marshy areas and fresh ponds due to its taller height and good culm strength. It is also stringently photosensitive maturing in January month, which enables its safe harvest and threshing when rainfall ceases to occur in these islands. This landrace expressed the “open floret” trait, which is documented as a novel biological feature with breeding ramifications in self-pollinated cereals such as rice (40), and it is imperative to note that all the 20 lines under investigation exhibited open floret tendency. Due to this unique feature and novel trait for hybrid breeding, this germplasm has also been registered in the Indian national genebank (IC0613963, INGR15014). Due to its luxuriant and clumpy growth of C14-8, a smaller number of seedlings for planting per unit area is required, which saves labor as well as seed rate. It has medium bold non-chalky grains that do not break even due to conventional hulling structures and has palatable and non-sticky cooked rice. This variety fits well under these conditions due to minimal management needs including less work involved for transplanting, weeding, and cultural operations, which also suits the organic mandate of the Islands. It has also been observed to have a strong resurgence and kneeing ability after disease and pests and cyclonic stresses. Additional straw due to its tall stature is also used for cattle feeding (after lopping during early stages and after threshing) or plowing into the soil, traditional houses thatching, barnyard covers, and mushroom cultivation. C14-8 attains a quite tall stature (approximately 200 cm height), matures very late (180 days), has 7–8 tillers/plant with longer panicles (30 cm), good spikelet fertility (75%) with short bold grains having test weight (1,000 grains) of approximately 26 g.

Our interaction with some octogenarian farmers revealed that conventionally C14-8 had yellow-colored grains but subsequently other grain color types got intruded in it, although the red grain type is traditionally perceived as more adaptable to swampy/marshy areas. Recently, it has been found that C14-8 grains also possess higher contents of zinc (Zn ~30 ppm) than the standard check varieties Swarna (14 ppm) and IR64 (15 ppm). Similarly, C14-8 grains showed a higher amount of iron (Fe ~16 ppm) than Swarna (14 ppm) and IR64 (9 ppm; our unpublished data). Understandably, all these traits might have accorded socioeconomic popularity to this landrace and thus served the food and nutritional requirements of the geographically isolated Andaman and Nicobar Islands for a long time.

### 2.3. Plant materials, characterization, and evaluation

Approximately 150 panicles showing grain husk color variation in C14-8 landraces were collected from diverse fields in Andaman districts at the time of maturity in the month of January 2012. During the subsequent years (2012–2014), all these panicle-to-row progenies were grown and evaluated for both qualitative and quantitative traits. Based on grain yield and grain husk color, 20 lines were chosen from these panicle-to-row progenies so that five representative lines from each of the four grain husk color types were selected for further investigation. The 20 lines were classified into four groups, such as Group I: brown husk with white apiculus, Group II: yellow husk with black apiculus, Group III: yellow husk with yellowish apiculus, and Group IV: golden furrows on brown husk with black apiculus (Table 1; Figures 2B,C). These four groups, henceforth, will be referred to as basic classification groups. During 2012 and 2014, these 20 selections of C14-8 were characterized and evaluated in a randomized block design (RBD) with three replications at Bloomsdale Farm, ICAR-CIARI, and Port Blair for morphological markers, such as basal leaf sheath color, leaf sheath anthocyanin, stem length, anthocyanin pigment on nodes, apiculus color, grain husk color, panicle secondary branching, leaf senescence, and decorticated grain color, and the data on the recorded traits were averaged across 2 years, which is more representative. The lines were also evaluated for quantitative traits such as plant height (PH, cm), days to 50% flowering (DF), ear bearing tillers per plant (EBT), panicle length (PL, cm), 1,000-grain weight (TGW, gm), grain length (GL, mm), grain width (GW, mm), grain yield (GY, t/ha), brown rice recovery (BRR, %), milled rice recovery (MRR, %), and head rice recovery (HRR, %), as mentioned in Table 2. The chemical test to confirm whether C14-8 belongs to *indica* or *japonica* sub-specific group revealed C14-8 to be *japonica* type due to yellow grain husk color retention as per the method of Sanni et al. (35).

**Table 1:** Morphological descriptors of 20 lines derived from C14-8 rice population*.

Group	Genotypes	Leaf sheath intensity of anthocyanin color	Stem length	Anthocyanin color on nodes	Apiculus color	Grain husk color	Panicle: secondary branching	Leaf: senescence	Decorticated grain: color
I	C14-8-11-1	Absent	V. long	Present	White	Brown husk	Clustered	Light	White
	C14-8-11-30	Absent	V. long	Present	White	Brown husk	Clustered	Light	Light red
	C14-8-11-91	Absent	Long	Present	White	Brown husk	Clustered	Medium	White
	C14-8-11-92	Absent	Long	Present	White	Brown husk	Clustered	Medium	White
	C14-8-11-93	Absent	Long	Present	White	Brown husk	Clustered	Medium	White
II	C14-8-11-31	Present	Long	Absent	Purple	Yellow husk	Strong	Medium	White
	C14-8-11-32	Present	Long	Absent	Purple	Yellow husk	Strong	Medium	White
	C14-8-11-43	Present	V. Long	Absent	Purple	Yellow husk	Strong	Medium	White
	C14-8-11-59	Present	V. Long	Absent	Purple	Yellow husk	Strong	Medium	White
	C14-8-11-60	Present	V. Long	Absent	Purple	Yellow husk	Strong	Medium	White
III	C14-8-11-61	Absent	Long	Absent	Yellowish	Yellow husk	Clustered	Medium	White
	C14-8-11-90	Absent	Long	Absent	Yellowish	Yellow husk	Clustered	Medium	White
	C14-8-11-108	Present	Long	Absent	Yellowish	Yellow husk	Clustered	Medium	White
	C14-8-11-113	Present	Long	Absent	Yellowish	Yellow husk	Clustered	Medium	White
	C14-8-11-143	Present	Long	Absent	Yellowish	Yellow husk	Clustered	Medium	White
IV	C14-8-11-114	Present	Long	Absent	Yellowish	Golden furrows on brown husk	Clustered	Medium	White
	C14-8-11-115	Present	Long	Absent	Black	Golden furrows on brown husk	Clustered	Medium	White
	C14-8-11-116	Present	Long	Absent	Black	Golden furrows on brown husk	Clustered	Medium	White
	C14-8-11-117	Present	Long	Absent	Black	Golden furrows on brown husk	Clustered	Medium	White
	C14-8-11-118	Present	Long	Absent	Black	Golden furrows on brown husk	Clustered	Medium	White

* Classification method: Shobha et al. (41).

**Table 2:** Range and statistical parameters for agro-morphological and biochemical traits of C14-8 derived lines.

Trait	Range	Mean ± std. error	Variance	Coeff. var
PH	190.0–213.0	202.6 ± 1.3	35.5	2.9
DF	174.0–179.0	176.6 ± 0.3	2.6	0.9
EBT	7.0–9.0	7.3 ± 0.1	0.3	7.9
PL	28.0–33.0	29.7 ± 0.3	1.6	4.3
TGW	23.1–27.4	26.46 ± 0.3	1.7	7.2
GL	7.7–8.8	8.2 ± 0.1	0.1	3.3
GB	2.9–3.4	3.1 ± 0.0	0.0	4.5
GL/GB	2.4–2.9	2.61 ± 0.1	0.1	3.9
GY	1.6–4.3	2.6 ± 0.2	0.5	27.6
BRR	70.4–78.8	75.4 ± 0.5	4.8	2.9
MRR	58.8–73.5	67.4 ± 0.8	11.9	5.1
HRR	58.1–71.1	63.5 ± 0.9	16.4	6.4
AC	6.4–13.0	9.3 ± 0.3	1.7	14.0
RSC	1.7–2.6	2.0 ± 0.0	0.0	10.9
TSC	50.8–77.3	74.1 ± 1.6	55.4	10.0
GC	3.3–3.9	3.7 ± 0.0	0.0	4.2
AD	4.0–6.0	5.0 ± 0.1	0.1	7.6
PC	14.2–18.2	16.5 ± 0.2	0.8	5.4
FC	2.4–6.1	3.4 ± 0.2	0.9	27.7
AnC	−0.2-1.2	0.5 ± 0.1	0.1	64.4
ASA1	72.4–92.9	88.7 ± 0.9	16.3	4.6
ASA2	72.4–93.6	89.1 ± 0.9	17.1	4.6

PH, Plant height (cm); DF, Days to 50% flowering; EBT, Ear bearing tillers per plant; PL, Panicle length (cm); TGW, 1,000-grain weight (gm); GL, Grain length (mm); GW, Grain width (mm); GY, Grain yield (t/ha); BRR, Brown Rice Recovery (%); MRR, Milled rice recovery (%); HRR, Head Rice Recovery (%); AC, Amylose content (mg/100 mg); RSC, Reducing sugar content (mg/100 mg); TSC, Total sugar content (mg/100 mg); GC, Gel consistency; AD, Alkali digestibility; PC, Phenolic content (mg/100 mg); FC, Flavonoid content (mg/100 mg); AnC, Anthocyanin content (mg/100 mg); ASA1, % Antioxidant scavenging activity/1 g by DPPH; ASA2, % Antioxidant scavenging activity/1 g by ABTS.

The classification for stem length was carried out as per National Guidelines for the conduct of tests for distinctness, uniformity, and stability (DUS) published by ICAR-Indian Institute of Rice Research (IIRR), Hyderabad, India. Therefore, the following scale was followed for stem length: very short (<91 cm), short (91–110 cm), medium (111–130 cm), long (131–150 cm), and very long (>150 cm). Next, when panicles have relatively more secondary branches, it is classified as “strong.” When panicles have a greater number of tertiary branches, it is classified as “clustered.” Furthermore, leaf senescence is classified as “light” (observed only in two selections) and medium (18 selections) as per leaf color appearance at maturity according to Shobha et al. (41).

### 2.4. Estimation of biochemical traits

Rice starch (complex carbohydrate) has attracted attention for its use in foods, extruded products, soups, and dressings due to its small size of starch granules, neutral taste, and soft mouth feel. Similarly, the market value of rice is influenced by its cooking qualities (amylose content, gel consistency, and alkali spreading value) apart from differential amounts of phenolics, flavonoids, antioxidants, head rice yield, etc.

#### 2.4.1. Amylose content

Amylose content and total and reducing sugar were determined for all 20 single-panicle progenies using the standard protocol described by Sadasivam and Manickam (42).

#### 2.4.2. Alkali digestibility

A duplicate set of six whole-milled kernels without cracks was selected and placed in a plastic box (5 cm × 5 cm × 2.5 cm). Approximately, 10 mL of 1.7% (0.3035 M) potassium hydroxide (KOH) solution was added. The samples were arranged to provide enough space between kernels to allow for spreading. The boxes were covered and incubated for 23 h in a 30°C oven. Starchy endosperm was rated visually based on a seven-point numerical spreading scale (43).

#### 2.4.3. Gel consistency

The gel consistency (GC) is based on the consistency of a cold 4.4% milled rice paste in 0.2 M KOH (44). The GC was measured by the length of the cold gel in the culture tube held horizontally for 0.5 to 1 h. The GC of rice with less than 24% amylose is usually soft. The test separated high-amylose rice into three categories as follows:

1. Very flaky rice with hard GC (length of gel 40 mm or less).
2. Flaky rice with medium GC (length of gel 41 to 60 mm).
3. Soft rice with soft GC (length of gel more than 61 mm).

#### 2.4.4. Total phenolic content

Total phenolic content in fresh samples was determined with the Folin–Ciocalteu reagent by the method described by Singleton et al. (45), with some modifications. In brief, 0.2 mL of a sample extract (1 mg/mL) was mixed with 1 mL of a 10-fold dilution of the Folin–Ciocalteu reagent and 0.8 mL of 15% (w/v) sodium bicarbonate solution and allowed to stand at room temperature for 30 min. The absorbance was measured at 765 nm, using a UV–visible spectrophotometer (Elico SL-164, Elico Ltd., Hyderabad, India), and total phenolic content was expressed as gallic acid equivalent (mg/100 g fresh weight).

#### 2.4.5. Total flavonoid content

Total flavonoid content was determined by a colorimetric method (46). In brief, 0.5 mL extracts were added to 15 mL polypropylene conical tubes containing 2 ml ddH_2_O and mixed with 0.15 mL of 5% NaNO_2_. After reacting for 5 min, 0.15 mL of 10% AlCl_3_.6H_2_O solution was added. After another 5 min, 1 mL of 1 M NaOH was added. The reaction solution was mixed well and kept for 15 min, and the absorbance was determined at 415 nm. Quantification was performed using Rutin as standard, and the results were expressed as milligrams of Rutin equivalent (mg RE) per 100 g of flour weight.

#### 2.4.6. Total anthocyanin content

Total anthocyanin content was analyzed using the pH differential method (42). Cyanidin-3-glucoside was used as the reference, and results were expressed as mg of cyanidin-3-glucoside equivalent per 100 g fresh weight, using the following equation: TAC = DA × MW × DF × 1,000)/ε where TAC is total anthocyanin content, DA is difference in optical density (OD) value of extract at pH 1.0 and pH 4.5, MW is the molecular weight of cyanidin-3-glucoside, DF is dilution factor, and ε is molar absorbance coefficient of cyanidin-3-glucoside.

#### 2.4.7. 2,2-diphenyl-1-picrylhydrazyl activity

Total antioxidant activity was obtained by the 2,2-diphenyl-2-picrylhydrazyl (DPPH) method (47) with some modifications. The working solution of DPPH was freshly prepared by diluting 3.9 mg of DPPH with 95% ethanol to get an absorbance of 0.856 ± 0.05 at 517 nm. The different concentration of the extract was mixed with 1.5 mL of working DPPH, and the absorbance of the mixture was immediately measured spectrophotometrically after 10 min. The total antioxidant activity of the extracted rice was expressed as mg BHA/g sample equivalent, obtained from the calibration curve.


$$\%\mathrm{Inhibition of DPPH radical}=\left(A_{\mathrm{control}}-A_{\mathrm{sample}}/A_{\mathrm{control}}\right)\times 100.$$

where A_control_ is the absorbance of the control (without extract), and A_sample_ is the absorbance in the presence of the extract/standard.

#### 2.4.8. 2,2′-azino-bis (3-ethylbenzothiazoline-6-sulphonic acid) activity

The total antioxidant capacity was determined by a colorimetric method (48) with a little modification. First, ABTS^**+**^ solution was prepared and adjusted with pH 0.784 ± 0.01 with 80% ethanol at 734 nm. Then, 3.9 mL ABTS^**+**^ cation solution was added to 1 mL of extracts and mixed thoroughly. The mixture was incubated for 6 min at room temperature and tested for absorbance at 734 nm. The results were expressed in terms of Trolox equivalent antioxidant capacity (TEAC, mM Trolox equivalents per 100 g dry weight).


$$\%\mathrm{Inhibition of ABTS radical}=\left(A_{\mathrm{control}}-A_{\mathrm{sample}}/A_{\mathrm{control}}\right)\times 100$$

### 2.5. Genomic DNA extraction and molecular profiling with SSR markers

The total genomic DNA was extracted from leaves of selections and the original mixed population of C14-8 as described by Murray and Thompson (49). PCR amplification was performed with 50 pairs of HvSSR markers spanning all 12 chromosomes (50) using a thermal cycler (Bio-Rad, United States). The thermal profile of PCR reactions was set as follows: initial denaturation at 94°C for 5 min followed by 35 cycles of denaturation at 94°C for 1 min, annealing temperature of 55°C for 1 min, extension at 72°C for 2 min, and a final extension at 72°C for 7 min. The PCR reaction volume of 10 μL was constituted using 50 ng genomic DNA, 1 × PCR buffer, 0.1 mM dNTP, 5 pmole each of forward and reverse primers, 2 mM MgCl_2_, 0.2 units of Taq polymerase, and nuclease-free water. The amplified product was resolved in 3% metaphor agarose gel in 1X TAE buffer along with a 1 kb ladder for 1 h at 80 V (51). The gel was analyzed using a gel documentation system (Bio-Rad, United States).

#### 2.5.1. Allele scoring

Different markers amplified a different number of bands for each genotype. Image Lab software (Bio-Rad, United States) was used to determine the size of the bands based on differential migration relative to standard molecular weight markers (50–1,000 bp ladder, Thermo-Scientific, United States). Alleles were numbered from one to as many alleles as obtained for a particular marker x genotype combination based on their molecular weight from lowest to highest. The presence of a band for a particular allele was scored as “1,” while the absence was scored as “0.” The most polymorphic markers were determined based on their PIC value of ≥0.70 and polymorphic alleles of ≥6 as per Babu et al. (52).

## 3. Data analysis

Mean data of agro-morphological and biochemical traits were used for calculating descriptive statistics, cluster analysis, principal component analysis (PCA), and character association studies. PCA and character associations were carried out using the software package PAST [paleontological statistics software package for education and data analysis (53)]. Clustering of genotypes was performed based on the Euclidean distance and Ward’s method, and the analysis was performed using SPSS 17Q15 software (IBM, United States), and the heatmap was generated using the R software package “gplots” (54). SSR marker-based clustering of genotypes based on the Euclidean distance and neighbor-joining method was performed using the software package PAST (53).

The Polymorphic Information Content (PIC) of each SSR marker was estimated using the following formula as per Botstein et al. (55).


$$1-\left(\overset{n}{\underset{i=1}{\sum }}p_{i^{2}}\right)-\overset{n-1}{\underset{i-1}{\sum }}\overset{n}{\underset{j=i+1}{\sum }}2p_{i^{2}}p_{j^{2}}$$

where P*_i_* = frequency of ith allele, and P*_j_* = frequency of jth allele. Alleles whose frequency is less than 0.05 were considered rare alleles (34).

## 4. Results

### 4.1. Characterization for qualitative traits

A discernible variation was recorded for the qualitative traits in C14-8 selections especially for grain husk color (Figures 2B,C; Table 1). All genotypes exhibited light purple colored basal leaf sheath except for two selections such as 1 and 30 from group I, which showed distinct green basal leaf sheath color as well as light red decorticated grains. Thirteen accessions in group II exhibited higher intensity of anthocyanin coloration in their leaf sheaths, which was absent in the remaining selections. Only group I selections showed anthocyanin coloration on nodes, which was absent in all remaining groups. Out of the 20 selections, five were classified as very long-stemmed and the remaining 15 as long stemmed. Selections of groups I and II had white and purple apiculi, respectively, while groups III and IV exhibited yellowish and black apiculi, respectively. Grains of groups II and III were yellow husked, and grains of groups I and IV were brown husked, while the latter was golden furrowed. Selections of group II exhibited strong secondary branching while selections in the remaining groups had clustered secondary branching. Leaf senescence was medium in all except for two selections 1 and 30 from group I, which showed light leaf senescence. Similarly, the decorticated grain color of all selections was white except for selection C14-8-11-30 from group I, which expressed light red color.

### 4.2. Descriptive statistics and character associations

Our study assumes cultural and agro-evolutionary significance because the diverse grain color selections descended from a common ancestral traditional landrace, which has been on-farm maintained under the geographical isolation of the Andaman and Nicobar Islands. It is also imperative to understand the inter-trait correlation dynamics in such biologically unique populations for their utilization during indirect selection for trait improvement.

**Figure 3:**
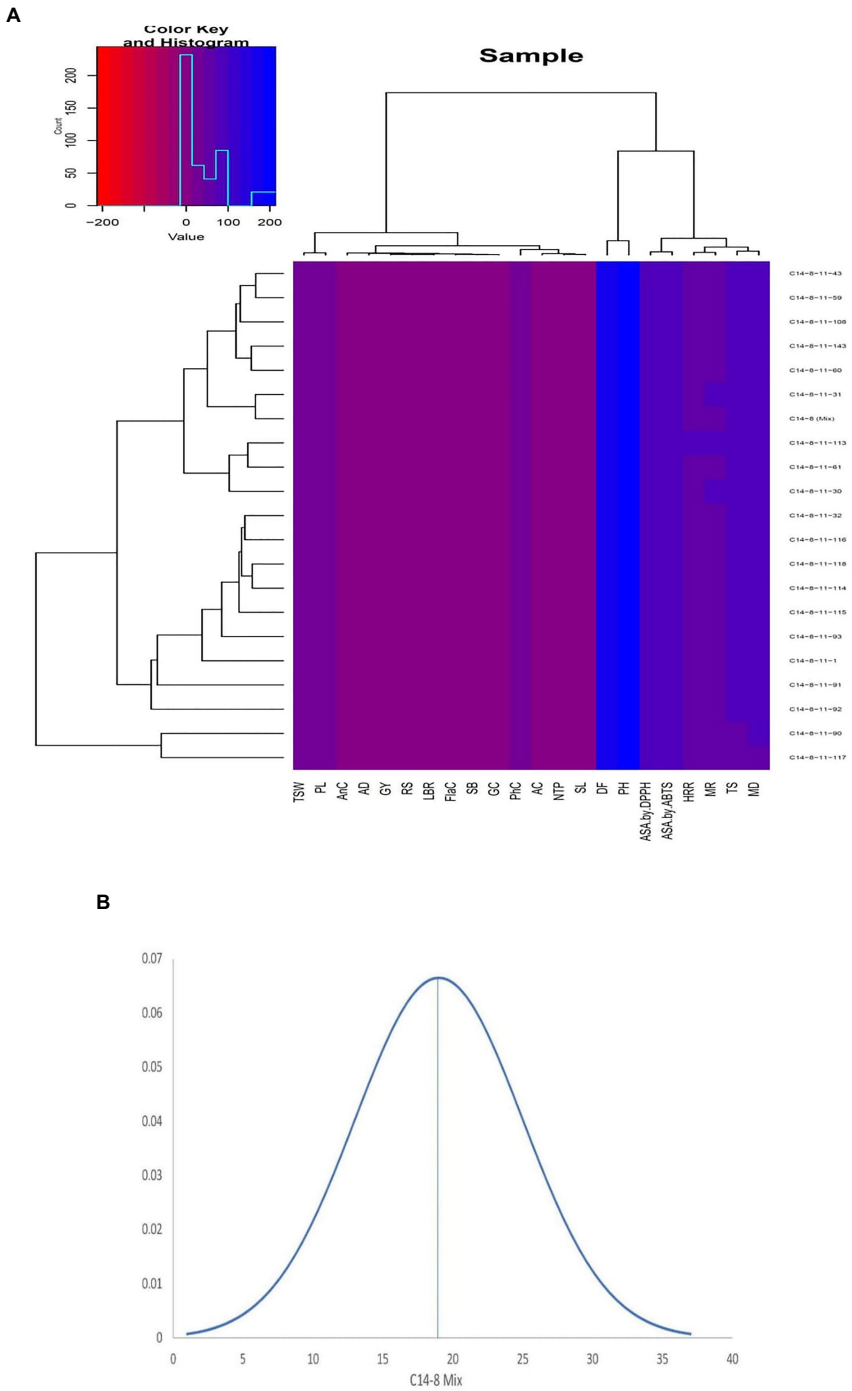
**(A)** Agro-morphological and bio-chemical traits heatmap of the 21 intra-varietal C14-8 selections in rice based on Euclidean distances. **(B)** Normal distribution curve of the means of all traits of C14-8 selections with respect to C14-8 mix population.

In general, the agro-morphological traits studied displayed a low coefficient of variation (%) except for the economically important GY (27.6), as shown in Table 2; Figure 3A, 4A,B. Among the biochemical traits, FC (27.7) and AnC (64.4) were highly variable, and traits AC (14.0), RSC (10.9), and TSC (10.0) were moderately variable while the remaining traits were least variable. It was also interesting to note that all the 22 traits using their mean values followed the normal distribution pattern, wherein the C14-8 mixed population possessed the central value (Figure 3B), indicating the polygenic nature of the population. Character association study of significant correlations revealed that PH was negatively correlated with four morphological traits of grain, such as TGW (−0.41), BRR (−0.44), MRR (−0.55), and HRR (−0.45), as shown in Table 3. DF was positively correlated with GY (0.49) and FlaC (0.47) but negatively correlated with RSC (−0.41). EBT was positively correlated with GL (0.67) and GB (0.67). TGW was found negatively correlated with RSC (−0.40). GL was positively correlated with GB (0.46), but GB was negatively correlated with GL/GB (−0.67) and FlaC (−0.41). BRR was positively associated with MRR (0.40), and in turn, MRR was positively correlated with HRR (0.75). AC was positively correlated with GC (0.41) and PhC (0.42). However, a negative correlation was observed between PhC and FlaC (−0.55; Table 3).

**Figure 4:**
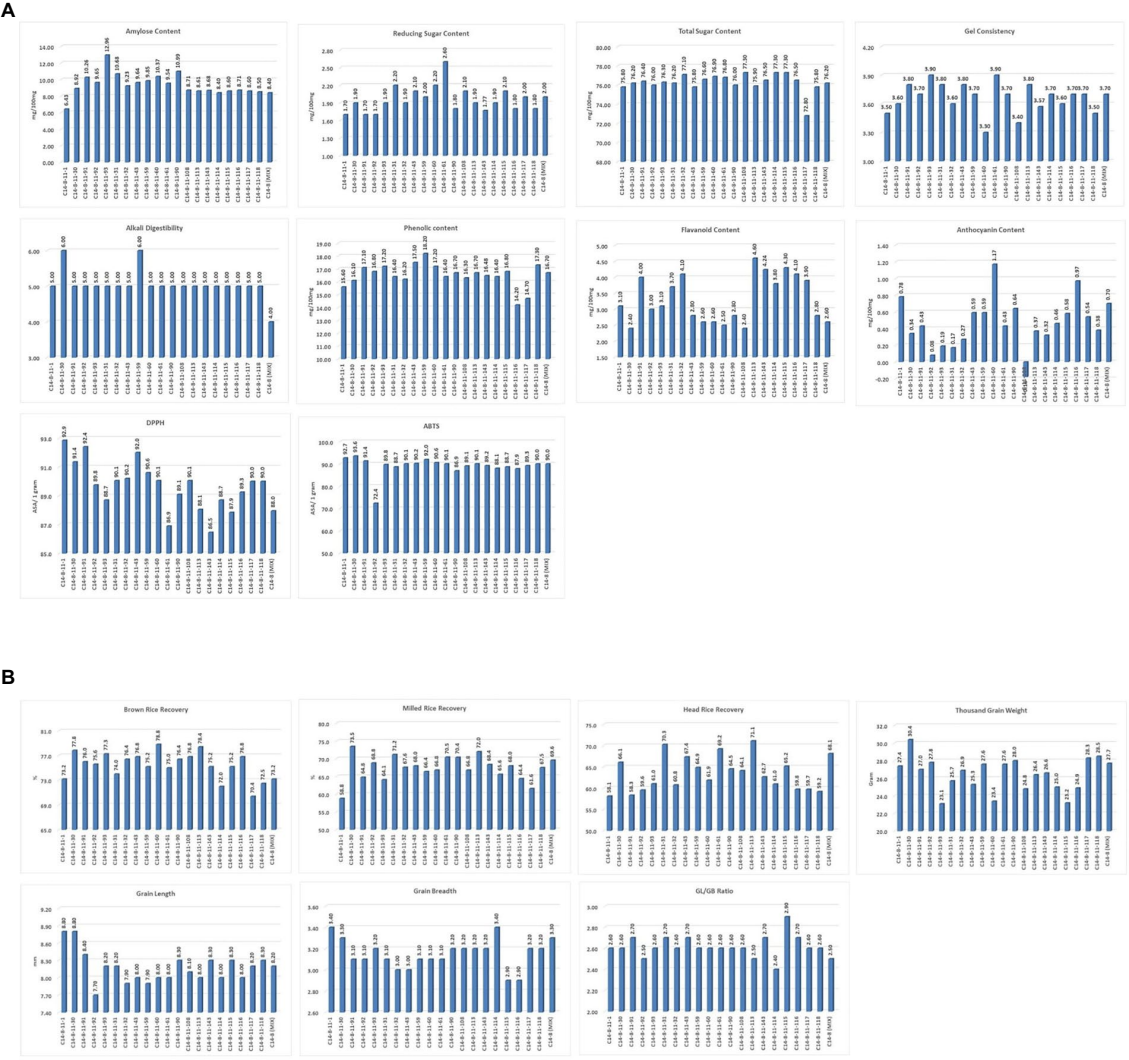
**(A)** Intra-Varietal variation for biochemical and cooking parameters in C14-8 selections. **(B)** Intra-Varietal variation for grain and milling parameters in C14-8 selections.

**Table 3:** Pearson’s correlation coefficients among morphological and biochemical traits in C14-8 population.

	PH	DF	EBT	PL	TGW	GL	GB	GL/GB	GY	BRR	MRR	HRR	AC	RSC	TSC	GC	GC	AD	PhC	FlaC	AnC	ASA by DPPH
PH	1.00																					
DF	0.12	1.00																				
EBT	−0.09	0.05	1.00																			
PL	0.06	0.00	0.16	1.00																		
TGW	−0.41*	−0.14	0.33	0.01	1.00																	
GL	−0.06	0.12	0.67*	0.19	0.32	1.00																
GB	−0.15	0.02	0.47*	0.28	0.38	0.46*	1.00															
GL/GB	0.19	0.10	0.00	−0.23	−0.31	0.25	−0.67*	1.00														
GY	0.00	0.49*	−0.03	−0.05	−0.13	0.00	0.28	−0.22	1.00													
BRR	−0.44*	−0.22	−0.07	0.09	−0.30	−0.13	−0.37	0.16	−0.06	1.00												
MRR	−0.55*	−0.23	0.11	0.00	0.24	−0.14	−0.12	−0.04	−0.27	0.40*	1.00											
HRR	−0.45*	−0.31	−0.04	−0.03	0.01	−0.12	−0.08	0.04	−0.15	0.25	0.75*	1.00										
AC	0.08	−0.09	−0.19	0.25	−0.26	−0.33	−0.32	0.10	−0.11	0.33	0.22	0.21	1.00									
RSC	−0.07	−0.41*	−0.24	−0.31	−0.40*	−0.20	−0.18	0.12	−0.33	0.24	0.18	0.33	0.16	1.00								
TSC	0.18	−0.19	0.18	−0.11	−0.33	−0.12	−0.13	0.06	−0.11	0.29	0.11	0.10	0.05	−0.12	1.00							
GC	0.11	0.14	−0.04	0.30	−0.31	−0.11	−0.08	−0.06	0.13	0.26	0.15	0.11	0.41*	0.12	−0.20	1.00	1.00					
AD	−0.23	0.03	0.38	0.03	0.10	0.21	−0.15	0.24	−0.03	0.36	0.17	0.06	0.10	−0.03	0.04	−0.06	−0.06	1.00				
PhC	−0.26	−0.29	−0.14	−0.09	−0.05	−0.13	−0.01	0.01	−0.07	0.23	0.40*	0.32	0.42*	0.12	0.14	0.05	0.05	0.16	1.00			
FlaC	0.35	0.47*	−0.02	−0.06	−0.26	−0.13	−0.41*	0.29	0.22	−0.02	−0.19	−0.17	−0.17	−0.31	0.03	0.20	0.20	−0.14	−0.55*	1.00		
AnC	0.05	−0.05	−0.14	−0.17	0.12	0.02	−0.14	0.11	−0.38	−0.20	−0.26	0.00	−0.17	0.02	−0.09	−0.21	−0.21	−0.12	−0.13	0.20	1.00	
ASA by DPPH	−0.12	−0.24	−0.16	0.40	0.13	−0.03	0.16	−0.20	−0.30	−0.12	0.09	0.21	−0.22	0.16	−0.19	−0.31	−0.31	0.17	−0.15	−0.24	0.10	1.00
ASA by ABTS	−0.03	−0.09	0.29	−0.20	0.00	0.58	0.19	0.23	−0.11	0.07	−0.10	0.16	−0.13	0.23	−0.05	−0.10	−0.10	0.20	0.02	−0.06	0.27	−0.06

PH, Plant height (cm); DF, Days to 50% flowering; EBT, Ear bearing tillers per plant; PL, Panicle length (cm); TGW, 1,000-grain weight (gm); GL, Grain length (mm); Grain width (mm); GY, Grain yield (t/ha); BRR, Brown Rice Recovery (%); MRR, Milled rice recovery (%); HRR, Head Rice Recovery (%); AC, Amylose content (mg/100 mg); RSC, Reducing sugar content (mg/100 mg); TSC, Total sugar content (mg/100 mg); GC, Gel consistency; AD, Alcohol digestibility; PC, Phenolic content; FC, Flavonoid content (mg/100 mg); AnC, Anthocyanin content (mg/100 mg); ASA by DPPH, % Antioxidant scavenging activity/1 g by DPPH; ASA by ABTS, % Antioxidant scavenging activity/1 g by ABTS.

^*^Significance at 0.05 probability.

### 4.3. Diversity based on agro-morphological and biochemical traits

**Figure 5:**
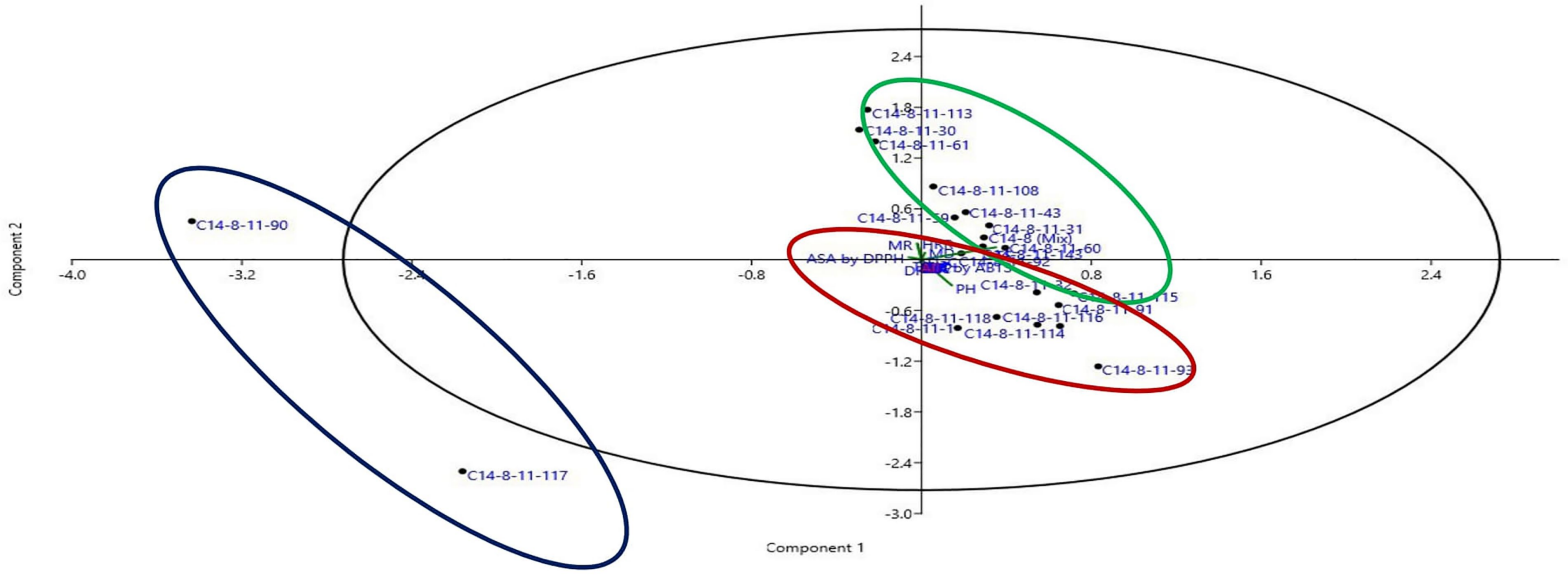
Two-dimensional scatter plot of PCA ordination using two most informative axis of 21 selected C14-8 rice lines obtained through PCA analysis.

Data on agro-morphological and biochemical traits of 20 C14-8 selections and the original C14-8 population were subjected to cluster analysis based on the Euclidean distance-based genetic dissimilarity, and the heatmap generated is presented in Figure 3A. It grouped these 21 rice genotypes into three major clusters with 10 (C14-8-11-43, C14-8-11-59, C14-8-11-108, C14-8-11-143, C14-8-11-60, C14-8-11-31, C14-8 mix, C14-8-11-113, C14-8-11-61, C14-8-11-30), 9 (C14-8-11-32, C14-8-11-116, C14-8-11-118, C14-8-11-114, C14-8-11-115, C14-8-11-93, C14-8-11-1, C14-8-11-91, C14-8-11-92), and 2 (C14-8-11-90, C14-8-11-117) genotypes in Clusters I, II, and III, respectively. Cluster I was mainly represented by the C14-8 selections belonging to groups II and III of our classification of these 20 genotypes on the basis of grain husk color and apiculus color, whereas, Cluster II was mainly represented by the C14-8 selections belonging to groups I and IV. The first five PCs (principal components) of PCA explained a significant proportion of the variation (90.95%) present in agro-morphological and biochemical traits (Table 4). The first two PCs explained 63.3% of the total variation with PC1 and PC2 explaining 35.44% and 27.91%, respectively. Traits such as TSC, PH, BRR, FlaC, AC, EBT, PhC, GL/GB, HRR, MRR, TSC, BRR, ASA by DPPH, TGW, PhC, ASA by 2,2′-azino-bis (3-ethylbenzothiazoline-6-sulphonic acid; ABTS), AC, AD, EBT, and RSC contributed to PC1 and PC2, respectively (Figure 5). Distribution of genotypes and in-turn classification of the same by plotting the first two PCs against one another resulted in a similar classification as that of cluster analysis with genotypes under Cluster I (green colored) and Cluster II (red colored), as shown in Figure 5. Here, we can see that most of the genotypes are clustered in green and red oval groups, and only two are in the blue oval groups and hence explained 63.3% of the total variation with PC1 and PC2.

**Table 4:** Percent variance of first five principal components for quantitative traits of 20 rice genotypes selected from C14-8 population.

Principal components	Eigenvalue	Variation (%)	Cumulative variation (%)	Characters involved
1	60.28	35.44	35.44	TSC, PH, BRR, FlaC, AC, EBT, PhC, GL/GB,
2	47.47	27.91	63.35	HRR, MRR, TSC, BRR, ASA by DPPH, TGW, PhC, ASA by ABTS, AC, AD, EBT, RSC
3	18.27	10.74	74.09	ASA by ABTS, BRR, HRR, EBT, GL, PhC, AnC, DF, GY, AD, FlaC, RS, GL/GB, GB, GC
4	17.14	10.07	84.16	ASA by DPPH, HRR, ASA by ABTS, PH, PL, MRR, RSC, TSC, AnC, AD, GL, GB, GL/GB
5	11.54	6.79	90.95	HRR, MRR, PH, AC, PhC, FlaC, GC, DF, RSC, GY, GL/GB,

PH, Plant height (cm); DF, Days to 50% flowering; EBT, Ear bearing tillers per plant; PL, Panicle length (cm); TGW, 1,000-grain weight (gm); GL, Grain length (mm); GB, Grain breadth (mm); GY, Grain yield (t/ha); BRR, Brown Rice Recovery (%); MRR, Milled rice recovery (%); HRR, Head Rice Recovery (%); AC, Amylose content (mg/100 mg); RSC, Reducing sugar content (mg/100 mg); TSC, Total sugar content (mg/100 mg); GC, Gel consistency; AD, Alcohol digestibility; PC, Phenolic content; FC, Flavonoid content (mg/100 mg); AnC, Anthocyanin content (mg/100 mg); ASA1, % Antioxidant scavenging activity/1 g by DPPH; ASA2, % Antioxidant scavenging activity/1 g by ABTS.

### 4.4. Cooking qualities and biochemical properties

In a bid to reveal the variability of C14-8 genotypes for quality attributes, physicochemical properties were studied and classified based on hierarchical cluster analysis. Cluster analysis is a statistical method to convert various characteristics of objects to quantitative measures also called similarity distance and correspondingly to cluster them at relatively close distances into a category. We hypothesized that cluster analysis can be used to categorize the progenies having similar properties into a single group. As shown in Table 5, the 20 selections are classified based on factors influencing cooking qualities, phytochemical content, and carbohydrate and amylose content. On an overall basis, selections C14-8-11-108, C14-8-11-114, and C14-8-11-115 have been separated into different groups.

**Table 5:** Cluster analysis of C14-8 variants based on cooking and nutritional quality parameters.

Parameters	Group-I	Group-II	Group-III	Group-IV	Group-V
Cooking qualities	C14-8-11-59	C14-8-11-93, C14-8-11-1	C14-8-11-30, C14-8-11-32, C14-8-11-115, C14-8-11-114, C14-8-11-116, C14-8-11-117, C14-8-11-118, C14-8-11-108	C14-8-11-91, C14-8-11-90, C14-8-11-31, C14-8-11-61, C14-8-11-92, C14-8-11-43, C14-8-11-60, C14-8-11-113, C14-8-11-143	-
Phytochemical content	C14-8-11-116	C14-8-11-1, C14-8-11-30, C14-8-11-91, C14-8-11-59	C14-8-11-92, C14-8-11-61, C14-8-11-108, C14-8-11-114, C14-8-11-115, C14-8-11-31, C14-8-11-93, C14-8-11-60, C14-8-11-118, C14-8-11-143	C14-8-11-32, C14-8-11-43, C14-8-11-117, C14-8-11-90, C14-8-11-113	-
Carbohydrate and amylose	C14-8-11-117	C14-8-11-93, C14-8-11-1	C14-8-11-30, C14-8-11-32, C14-8-11-118, C14-8-11-113, C14-8-11-116, C14-8-11-60	C14-8-11-91, C14-8-11-92, C14-8-11-31, C14-8-11-90, C14-8-11-43, C14-8-11-59, C14-8-11-61	C14-8-11-108, C14-8-11-114, C14-8-11-115, C14-8-11-143

### 4.5. Molecular diversity

**Figure 6:**
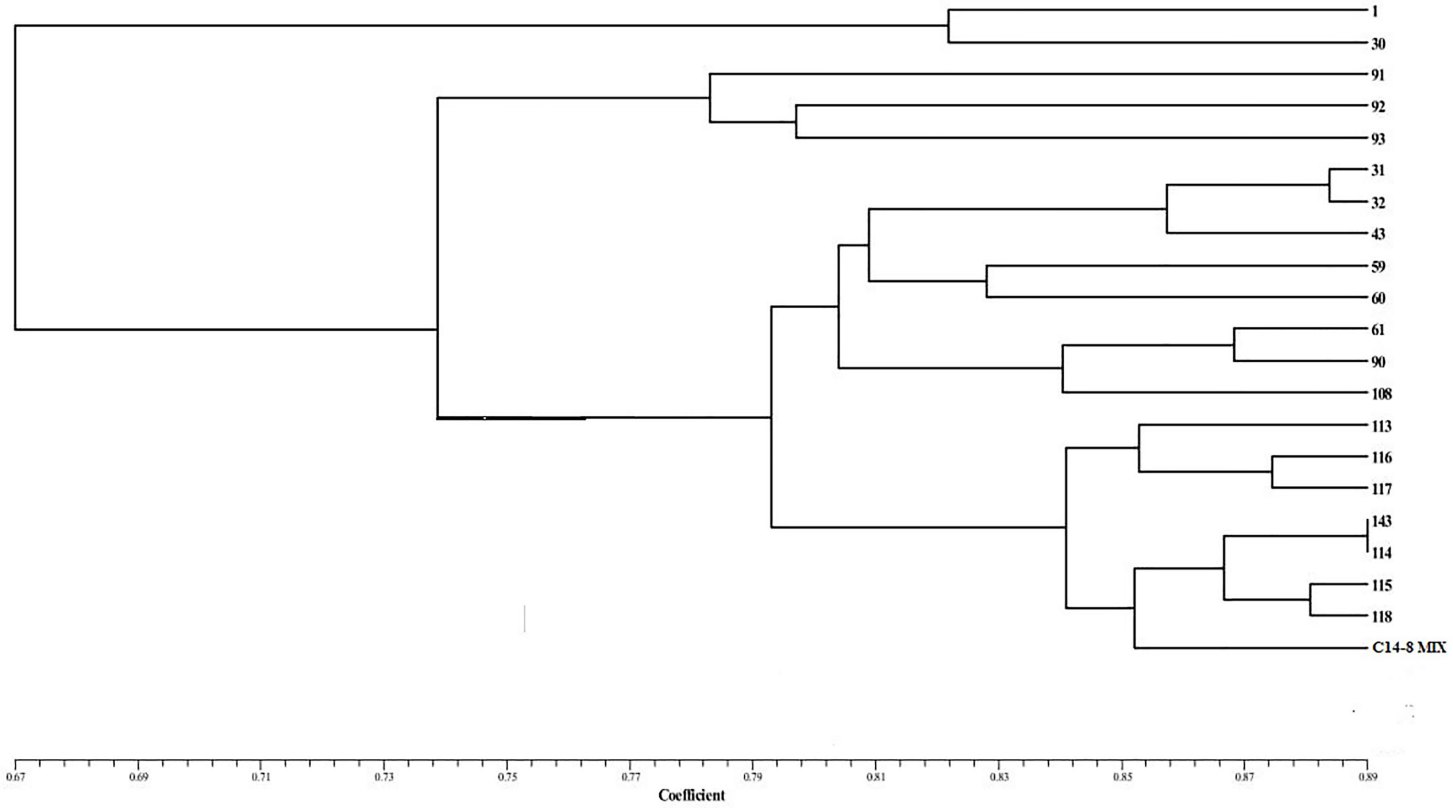
Dendrogram showing molecular diversity.

A total of 314 alleles were produced by 50 SSR markers, which ranged from 1 to 15 alleles per marker with an average of 6.28 (Supplementary Table 1). Out of 314 alleles, 64 alleles were found to be rare among the C14-8 selections. There were five markers with 0 PIC value. PIC values for the remaining markers ranged from 0.35 to 0.93, with an average of 0.67. Among the 50 HvSSR markers employed, 62% (31 markers) were most polymorphic with an average of 2.58 per linkage group. Among the linkage groups, chromosomes 2, 7, 9, and 11 harbored the most polymorphic loci (four markers) in the C14-8 population. On the other hand, none of them was present on chromosome 12. Clustering of genotypes based on Euclidean dissimilarity infers those selections of group II of the basic classification groups, which formed a separate group. Of the remaining, selections of group I and group IV formed separate clusters but interspersed with selections of group III. While three selections of group III aligned with group I, two selections got aligned with group IV. A similar classification was obtained except a genotype C14-8-11-59, which formed a solitary cluster when the dendrogram was constructed based on Jaccard’s similarity coefficient. Notably, the molecular clustering of the selections through SSR markers almost conformed to the grouping based on grain husk coloration (Figure 6).

## 5. Discussion

It is worth mentioning that the old landraces being grown and selected by farmers in their areas of adaptation for specific needs over the millennia have retained important traits and genes for crop improvement (9, 32). However, over the centuries, these traditional varieties have developed varying levels of heterogeneity (56, 57) plausibly due to manual admixtures as well as genetic events of recombination and mutations. In this context, it is interesting as well as imperative to understand the causes and types of intra-varietal variation for different phenotypic, biochemical, and molecular traits for genetic studies and breeding applications. Although C14-8 is grown as a single landrace variety, we observed typical open floret features and intra-varietal variation for the grain husk color, which prompted us to investigate the extent and pattern of diversity for different traits in these progenies and to project their implications in plant breeding. Although there are good numbers of studies on the diversity between varieties, pure lines, local cultivars, and/or landraces (31–36), systematic studies on intra-varietal variation are far more inadequate. Therefore, the dilution of genetic fidelity of landrace population due to inter-crossing over several decades of cultivation might reduce their productivity potential and popularity. Hence, the collection and genetic differentiation of landrace undertaken in the present study gain the importance to retain its on-farm diversity, varietal signatures, sustainability, and use as a donor for improvement of other varieties due to its salient traits, especially in light of socioeconomic requirements and emerging environmental challenges. In this context, Marone et al. (9) have also reviewed that traditional landraces have substantially contributed genes for stress adaptation in cereals.

This study perhaps reports a first-ever systematic and elaborate effort on the collection of diverse lines of a popular and promiscuous rice C14-8 followed by isolation, grouping, and identification of different types under geographical isolation of the Andaman Islands. The occurrence of distinct grain color types in the C14-8 population pool may be attributed to the manual admixture, spontaneous mutants, and/or occurrence of open florets, which indirectly permitted varying levels of intra-varietal as well as inter-varietal outcrossing resulting in a heterogeneous population undergoing natural selection over several decades. Here, it is pertinent to add that there is negligible chance of inter-varietal outcrossing due to significant phenological differences in flowering time between C14-8 (December month of the season) and other prominent varieties (before the November month of the season) owing to stringently photosensitive nature and significantly taller stature of tropical *japonica* C14-8 compared with all other *indica* rice varieties in these islands. In addition, the likely wide incompatibility at the *S5* locus (22) might have further reduced the probability of hybridization and recombination between C14-8 (tropical *japonica*) and local *indica* varieties. Therefore, it is quite probable that some spontaneous mutation or recombination at a few loci controlling grain color occurred in this open floret landrace, which could have floated in the population to cause intra-varietal variation. In support of our findings, according to Fujino et al. (58), while studying the ancestral implications of a Japanese rice variety, Kitaake has also concluded that the fast accumulation of pre-existing mutations in landraces might facilitate the adaptability of rice varieties to the local regions.

For rice grain husk color, Liu et al. (59) have fine-mapped *Pa-6*, a dominant gene on chromosome 6 governing the purple color of the apiculus, leaf sheath, and pericarp. Saitoh et al. (60) further reported that anthocyanin coloration in rice caused by the interplay of three basic genes, C (chromogen), A (activator), and P (distributor), is not only a morphological marker but also implicated in understanding of rice domestication from wild to cultivated type. Hence, our study was an endeavor to delve into these myriads and quantify genetic alteration and agro-cultural adaptation over decades in a promiscuous landrace population C14-8 under natural isolation of geographical remoteness. Similar to our attempt, a traditional Thai rice landrace Bue Chomee exhibited significant genetic differentiation among Karen villages as revealed by microsatellite markers (61). Due to common genetic background but variation for a few loci, the landraces can also serve as ideal mapping populations for molecular mapping of useful traits. While the process of domestication has reduced the stigma exsertion rate and length of stigma and anther, it caused thicker and wider lemma and palea in cultivated varieties than the wild rice accessions (62). Therefore, a shift from outcrossing to selfing occurred during the domestication of cereal crops except for maize, pearl millet, and sorghum (63–65). C14-8 was also found to exhibit perennial nature due to a considerably longer growth duration of approximately 8 months and better regeneration after ratooning as also reported in wild rice accessions (66).

Open floret is a rare reproductive trait documented in self-pollinated cereals such as rice, which can permit inter-varietal and intra-varietal out-crossing resulting in genetic variation. However, this rare trait if present in the restorer or male sterile lines can also be a boon for achieving a higher seed set in hybrid rice breeding. We have previously reported the occurrence of this deviant floral feature in C14-8, which got approved for registration (IC0613963, INGR15014) in the Indian national genebank (40). The swelling of lodicules adjacent to the ovary causes flower opening in cereals at anthesis in autogamous cereals such as rice, wheat, and barley (67–69). Some studies associated with flower opening and ovary size in wheat indicated that the bigger the size of the ovary, the wider will be floret opening (70). It is also perceived that sometimes lack of self-pollination in self-pollinated cereals has influenced flower opening to facilitate cross-pollination for setting seed, wider genetic base, and adaptation (71). The association of purple apiculus with purple basal leaf sheath color in group II as found in our study was also reported earlier by Liu et al. (59).

It is important to mention that the diverse lines descended from a common ancestral traditional landrace, which has been on-farm maintained under the geographical isolation of the Andaman and Nicobar Islands. Furthermore, the genetic fidelity of the C14-8 population in relation to other rice varieties was also maintained because C14-8 is stringently photosensitive, extremely late flowering (150 days), and very tall (190 cm) rice varieties unlike all other photo-insensitive and early maturing rice varieties grown in these islands. Therefore, it is opined that the varying levels of diversification of traits recorded by us could be plausibly due to selection pressure and development of spontaneous variants/mutants arising in the C14-8 followed by the floating of the variants in the entire population facilitated by open floret nature.

The variation in grain yield and related traits is not only important for analysis but also equally important for grain milling and nutritional characteristics. Because the C14-8 rice variety has long served the remote and tropical warm and humid islands, which are perceived to be vulnerable to geo-climatic aberrations such as the devastating tsunami in 2004, dry spells, as well as frequent cyclonic storms. Furthermore, the island population requires to consume a cheaper source of calories such as rice nutritionally rich in terms of antioxidants, flavonoids, anthocyanins, etc., due to the tropical hot, radiant, and humid climate. Although we have found that C14-8 is nutritionally rich in terms of higher zinc and iron contents, it was imperative to understand the status and variation of these selections for nutritional parameters in the popular C14-8 rice. Interestingly, Eigenvalue revealed that most of the percentage genetic variation among the representative lines is explained by the invisible but important grain milling and nutritional traits.

Adaptation of japonicas to tropical islands has been revealed by Thomson et al. (23) who found most rice landraces belonging to *japonica* types in the isolated Island of Borneo, Indonesia. This region is also geographically and climatically near the tropical Andaman and Nicobar Islands where C14-8 tropical *japonica* rice has carved out its cultural niche among traditional rice farmers due to their resource constraints, especially during the initial years of settlement in these islands. Since C14-8 has been adapted for long and survived the biotic and abiotic pressures in the geographically isolated islands, efforts could be directed to fish out useful genes underlying multiple stress adaptation traits for their likely utilization for improving stress tolerance of rice under changing climate (9). It was also interesting to find that C14-8 selected lines in our study planted in a zinc deficient field exhibited differential symptoms to this abiotic stress (unpublished data), which also indicated the probable variation for zinc deficiency adaptation, which however needs further validation through systematic studies. Furthermore, intra-varietal variants could also serve as a good population for genetic and molecular mapping of useful traits due to near common genetic background. It is also important to know that the traits or combination of traits exhibited maximum contribution to the total genetic variation in C14-8. The information, thus, generated could be utilized for pinpointing responsible traits for direct selection from a farmer’s perspective or else for identifying donors for genetic improvement of such a population. It is, therefore, interesting to look for such genotypes in the germplasm pool, which possess superiority for a maximum number of agronomic and biochemical traits. C14-8-11-33, C14-8-11-113, and C14-8-11-93 were identified as superior lines based on milling and biochemical traits. Our study also assumes cultural and agro-evolutionary significance because the diverse grain color selections descended from a common ancestral traditional landrace, which has been on-farm maintained under the geographical isolation of the Andaman and Nicobar Islands.

Descriptive statistics revealed good variability for traits such as GY, AC, FC, and AnC. Similarly, variability for biochemical traits can also be exploited for the improvement in grain quality. Such association studies between various traits have also been conducted previously (72–74), and the useful and easily traceable associations could be employed as an indirect selection for crop improvement.

Furthermore, we were also curious to know if the classification for grain husk color conformed to the cluster grouping based on the agro-morphological, biochemical, and molecular markers. Cluster analysis (CA) and PCA based on agro-morphological traits and biochemical traits resulted in broadly two groups except for the two selections forming a separate cluster. One of the two groups includes group I and group IV selections of the basic classification group, and the other group includes group II and group III selections. CA and PCA based on agro-morphological and biochemical traits support the basic classification, although they were grouped solely based on grain husk and apiculus color. A “correlated response” between the quantitative and qualitative traits and/or non-deliberated selection for phenotypic similarity during the selection process might have led to this correspondence. On the other hand, SSR marker profile-based CA grouped selections of group II into a separate cluster, and group I and group IV selections formed separate groups but interspersed with the selections of group III. It means the variability present in groups I, II, and IV is adequately represented by 50 SSR markers to classify them into separate clusters. However, employing additional markers would have possibly resulted in a separate group III cluster.

The extent of genetic diversity and degree of gene flow is influenced by anthropogenic activities, farming practices, prevailing climatic conditions (75), and geographical isolation (61). It is also pertinent to add here that crop landraces may possess considerable trait richness due to genetic diversity because these represent an intermediate stage between wild species and cultivated crops, and therefore, these become a natural choice for crop improvement (25). In general, farmers are known for maintaining the purity and perpetuity of local varieties/landraces through various traditional practices (76). However, the pattern and extent of diversity in the C14-8 variety, particularly for distinct grain husk color as well as qualitative and quantitative traits are surprising. This leaves the question of whether the variety itself was a mixture of pure lines when originally introduced in the islands or if different pure lines might have been brought initially but eventually turned into a mixture later. One of the causes of high variation recorded in the tropical *japonica* C14-8 selections may be spontaneous mutation with an intended or unintended selection of rare alleles for different traits over the years. While working in 148 traditional and modern cultivars of *indica* and *japonica* rice, Hour et al. (21) in Taiwan have also observed that despite less variation among cultivars, *japonica* landraces exhibited higher genetic variation than *indica* landraces. In addition, since the C14-8 cultivar exhibits an open floret tendency, there is a high probability of outcrossing among rare variants for grain husk color and other cryptic traits. The promiscuous tendency in C14-8 predisposes it to constant introgression of different alleles from various landraces and/or varieties (76) albeit the stringent photosensitive nature and *japonica* status of this landrace, further reinforced by geographical isolation eliminates the probability of its genetic recombination with other rice varieties in these islands. Gao et al. (77) have also concluded that intra-varietal variation among landraces was more pronounced due to a greater number of diverse alleles, especially at farmers’ fields than the modern varieties.

Therefore, varying levels of diversification of traits recorded by us could be plausibly due to the genetic events of open floret-mediated recombination, mutations, and selection drift in C14-8 followed by selection pressure caused by climatic variations, marginal input conditions, and differing agronomic practices practiced by farmers’ groups across scattered island ecosystem. It may also be pertinent to add that the expression of this unique floral attribute of this landrace might be confined to the specific microclimatic conditions of the Andaman and its expression beyond its natural habitat needs to be validated. Furthermore, the economic benefits for the commercial hybrid industry if any arising out of the genetic transfer of this novel trait may also be shared with the custodian farming community of these islands who are on farms conserving the landrace for the past several decades to ensure “conservation through compensation.”

## 6. Conclusion

The intra-varietal genetic diversity revealed was through 22 agro-morphological and biochemical traits, and molecular markers can be attributed to either accumulated mutation coupled with intended or unintended human selection, which might have increased the frequency of rare alleles over time in the C14-8 population. The findings could be further useful in rice and other self-pollinated crops in understanding the evolutionary significance and relevance of naturally occurring promiscuous behavior from genomic and biological perspectives. Futuristically, it is also worthwhile to explore this population for genetic mapping of its agronomically adaptive traits because the individuals, although may be contrasting in few traits, share almost common genetic background. Although rice is a self-pollinated crop, rare landraces such as C14-8 due to open floret tendency may represent a heterogeneous gene pool having both agronomically favorable and unfavorable alleles, which need further validation. Our observations and findings could be a precursor to identifying, quantifying, and utilizing the intra-varietal genetic variation in self-pollinated landrace populations exhibiting allogamous behavior. The useful variability can be captured, fixed, and purified in the form of new varieties or can serve as a source of allele mining for climatic resilience and nutritional traits, especially for marginal areas. While the open floret trait in self-fertilizing cereals could be potentially useful for transfer in hybrid parental lines for achieving efficient hybrid seed production, it may pose bottlenecks for obtaining molecular signatures for variety identity and purity for practical purposes. In view of the above, the pure line selection varieties derived from such landraces might necessitate temporal and spatial isolation for maintaining their genetic fidelity for sustainable conservation and production system. Therefore, identification, purification, and popularization of agronomically and nutritionally superior selections from this landrace population will aid in the sustainable rice production and livelihood security of the island population in terms of healthy food and improved economic and social system without impacting the natural environment. This will lead to the food sustainability and nutritional security of a large population in the remote Andaman and Nicobar Islands.

## Data availability statement

The original contributions presented in the study are included in the article/Supplementary material, further inquiries can be directed to the corresponding authors.

## Author contributions

RG: conceptualization, methodology, project administration, and editing. PS: methodology (designed and conducted field experiment). KV: data analysis using spatial software. BR: molecular data analysis and original draft writing. KS: methodology and conducted lab experiment (molecular work). SS: methodology and conducted lab experiment (biochemical analysis). MS: methodology (molecular analysis using SSR markers). SZ: methodology (social interaction with farmers). KD: field experiment and collection of rice samples. SR: methodology, conducted field experiment, and validation of the data. JV: tabulation and data curation. SA: writing the original draft. SL: writing the original draft, reviewing, and editing. All authors contributed to the article and approved the submitted version.

## Funding

The authors thank the Director, ICAR-Central Island Agricultural Research Institute, Port Blair for financial support to the project HORTCARISIL201200100146. We are also thankful to the Department of Biotechnology (DBT), Government of India for the financial grant (BT/PR6575/AG11/106/892/2012) for the “Application of molecular markers under accelerated crop improvement programme.” We also acknowledge the funding support from the Bill and Melinda Gates Foundation and IRRI, Philippines for the “Stress tolerant rice for poor farmers of Africa and South Asia” project.

## Conflict of interest

The authors declare that the research was conducted in the absence of any commercial or financial relationships that could be construed as a potential conflict of interest.

## Publisher’s note

All claims expressed in this article are solely those of the authors and do not necessarily represent those of their affiliated organizations, or those of the publisher, the editors and the reviewers. Any product that may be evaluated in this article, or claim that may be made by its manufacturer, is not guaranteed or endorsed by the publisher.

## Footnotes

^1^ https://www.downtoearth.org.in/news/andamans-lakshwadeep-declared-hope-spots-by-iucn-42556
