# Supplementary material for: Harnessing intra-varietal variation for agro-morphological and nutritional traits in a popular rice landrace for sustainable food security in tropical islands
Source: Front Nutr. 2023 Feb 23;10:1088208. doi: 10.3389/fnut.2023.1088208 (PMC9995847; doi:10.3389/fnut.2023.1088208)
Supplement: Supplementary file 1 [file Table_1.DOCX]

Supplementary Table 1. List of markers with their PIC value, number of alleles and sequences of forward and reverse primer

| Sl. No. | Primer name | Chr. No. | * PIC Value | Position  (in Mb) | #  Alleles | MP | Forward primer | Tm °C | Reverse primer | Tm °C |
| --- | --- | --- | --- | --- | --- | --- | --- | --- | --- | --- |
| 1 | Nksrssr01_20380 | 1 | 0.65 | 20.38 | 4 | 1 | TTACACCTTGTGCCTAACA | 47 | CCTCCAGCGATAATGAAGTAG | 52.4 |
| 2 | Nksrssr01_20427 |  | 0.52 | 20.427 | 3 |  | GCTCCAAGGTTCCAAAGT | 48 | TTTCTCCCTCAATGTCCTC | 48.9 |
| 3 | Nksrssr01_27499 |  | 0.00 | 27.499 | 1 |  | GCCAACACGAACTCCCGAAGG | 58.3 | CTGCTCCGTCTGCATCGTCTGC | 60.4 |
| 4 | Nksrssr01_28113 |  | 0.44 | 28.113 | 4 |  | GACTCACTCGTCTCGTGG | 52.6 | CTGGCATCAACTTCTCATT | 46.8 |
| 5 | Nksrssr01_31482 |  | 0.75 | 31.482 | 6 |  | GACGGTGTCGGTGGGCTT | 54.9 | TCGCTTCTCCTTCTCCCT | 50.3 |
| 6 | Nksrssr01_38964 |  | 0.57 | 38.964 | 5 |  | GCGTCGTCTCCTCACTCT | 52.6 | ACAAAGCCCATAACAACATC | 47.7 |
| 7 | Nksrssr02_1078 | 2 | 0.84 | 1.078 | 9 | 4 | TTAGGCACAATAAACACAGA | 45.6 | GAGATGGGATGGAGGAGT | 50.3 |
| 8 | Nksrssr02_3423 |  | 0.85 | 3.423 | 11 |  | ATCTATTCAGGGTTCAGGGT | 49.7 | CCCAAGTTCGTTACAGCA | 48.0 |
| 9 | Nksrssr02_24589 |  | 0.90 | 24.589 | 12 |  | GCATCGATCTTAATCTGCCC | 51.8 | CATGGCCATCCCTAAAACAC | 51.8 |
| 10 | Nksrssr02_35496 |  | 0.85 | 35.496 | 9 |  | CTGCATCAATATAATTGCGA | 45.6 | GCTACTTACACCACCACCAT | 51.8 |
| 11 | Nksrssr03_5219 | 3 | 0.84 | 5.219 | 7 | 2 | TGACCACCTGACTATGATG | 48.9 | AATCTGTTGCCATTTCTT | 41.2 |
| 12 | Nksrssr03_5694 |  | 0.37 | 5.694 | 2 |  | CAAGAAACAAGAACATCAAG | 45.6 | AAAGTGGGAAAGAAAGAGAAA | 45.6 |
| 13 | Nksrssr03_5942 |  | 0.00 | 5.942 | 1 |  | ATTGACACGAAGAGGACAAA | 47.7 | GTGCCCGAGGTGAGTGAGT | 55.4 |
| 14 | Nksrssr03_26474 |  | 0.37 | 26.474 | 2 |  | CACACCAACTCACTCTTGAA | 49.7 | CCGTTTCGTCTATGTTCATT | 49.7 |
| 15 | Nksrssr03_20495 |  | 0.88 | 20.495 | 10 |  | GATTGAAAATTAGAGTTTGCAC | 47.4 | GATTGAAAATTAGAGTTTGCAC | 47.4 |
| 16 | Nksrssr04_558 | 4 | 0.93 | 0.558 | 15 | 3 | GACGGTGAGAGAAGAGGG | 52.6 | ATCCAAATCCAAACTCCAA | 44.6 |
| 17 | Nksrssr04_25723 |  | 0.83 | 25.723 | 8 |  | TACATTAGGTGAGCCATCGT | 49.7 | AAGAAAGCCGTTTAGGACA | 46.8 |
| 18 | Nksrssr04_34639 |  | 0.85 | 34.639 | 8 |  | ACAATCAAGAACAGAAATCCA | 46.5 | TCAGCGAGACATCACTTC | 48.0 |
| 19 | Nksrssr04_34658 |  | 0.60 | 34.658 | 6 |  | GAAATCAACTGTGTCCTCAAC | 50.5 | CTTCTCGCCTGTCTCTCA | 50.3 |
| 20 | Nksrssr05_3420 | 5 | 0.90 | 3.42 | 11 | 3 | ACGGTTTGGTAGGGTGTC | 50.3 | AGGGTGGCAGGGATGTAA | 50.3 |
| 21 | Nksrssr05_3620 |  | 0.79 | 3.62 | 7 |  | ACGGAGGGAGTAGGTCATT | 51.1 | TGTGTGGAAGAATAGAGAGTCA | 51.1 |
| 22 | Nksrssr05_9030 |  | 0.78 | 9.03 | 5 |  | GCAAGGAAGGGATTATTGA | 46.8 | GTAGGTGGAGAGGAGA | 45.9 |
| 23 | Nksrssr05_29392 |  | 0.87 | 29.392 | 10 |  | TGCGATGAACTAATGGTAA | 44.6 | GAAGGACGACGACAAAGAC | 51.1 |
| 24 | Nksrssr06_293 | 6 | 0.75 | 0.293 | 6 | 2 | AAGAGATGGCTGAGGAAGA | 48.9 | CGAGTAGACAAAGAAAGCAAA | 48.5 |
| 25 | Nksrssr06_535 |  | 0.82 | 0.535 | 8 |  | TAGGGAATCAGCGGTTAG | 48 | GCTCTCTTGTCCTTCTTCTTC | 52.4 |
| 26 | Nksrssr06_28038 |  | 0.61 | 28.038 | 5 |  | ATCAGTTCAGTTCCCGCA | 48 | AAGTTCGCATCAGCAAAG | 45.8 |
| 27 | Nksrssr06_30941 |  | 0.00 | 30.941 | 1 |  | TTAGTGCCTGTTTGTTTCCT | 47.7 | GAGCAAGTTAGTGTGGTCTTC | 52.4 |
| 28 | Nksrssr07_19349 | 7 | 0.73 | 19.349 | 6 | 4 | CAACTGACGGAGTGGGTG | 52.6 | TCGTGTCGTGTCTTTGTTT | 46.8 |
| 29 | Nksrssr07_22533 |  | 0.74 | 22.533 | 7 |  | TAGGGACGATGGACGAAC | 50.3 | ACTTGGAACGAGAGAGAGATT | 50.5 |
| 30 | Nksrssr07_23994 |  | 0.83 | 23.994 | 7 |  | TTCTAAACCACGAAGATAGCA | 48.5 | AGACTACAAACCAAACCAACC | 50.5 |
| 31 | Nksrssr07_27576 |  | 0.84 | 27.576 | 8 |  | GTAGACACCGACGACGAC | 52.6 | TCCACCTCCTTCAGCCAC | 52.6 |
| 32 | Nksrssr08_3246 | 8 | 0.91 | 3.246 | 11 | 3 | ATGCAATACAGCACACTCGC | 51.8 | CTTATGCTCTCATGGCTCCC | 53.8 |
| 33 | Nksrssr08_5992 |  | 0.82 | 5.992 | 7 |  | AGTCCTTGAGAGAGATTGGAC | 52.4 | CGTATCCTCATCATCTCTTTG | 50.5 |
| 34 | Nksrssr08_8753 |  | 0.83 | 8.753 | 7 |  | AATAGCAGTAGTACACCGGAA | 50.5 | GCGAAGGAAGGAGTAGTAAAT | 50.5 |
| 35 | Nksrssr08_10937 |  | 0.79 | 10.937 | 5 |  | CGTACATACTTGGGATGAGC | 51.8 | GCCATGATCAAAGCATTATT | 45.6 |
| 36 | Nksrssr09_3177 | 9 | 0.90 | 3.177 | 11 | 4 | GAGATTGGTGTGACCCTT | 48 | CCTACGGCTCCTGACATT | 50.3 |
| 37 | Nksrssr09_4172 |  | 0.88 | 4.172 | 9 |  | GCGATGACTTTGTTACTCT | 46.8 | GGCGGTTTAGGAGCGTTT | 50.3 |
| 38 | Nksrssr09_9921 |  | 0.77 | 9.921 | 6 |  | AGAATCACACACAGACACACA | 50.5 | CGCTGAGGAGACTATGACTTT | 52.4 |
| 39 | Nksrssr09_16935 |  | 0.76 | 16.935 | 6 |  | CGTGAAAGTGACGAGTTTCAGTCC | 57.4 | CGAAGTGAACATGGCAAACC | 51.8 |
| 40 | Nksrssr10_9 | 10 | 0.00 | 0.009 | 1 | 1 | TTCAGAACACACCAAACTGA | 47.7 | GATTGAAAGTGCATCTAGGC | 49.7 |
| 41 | Nksrssr10_17995 |  | 0.00 | 17.995 | 1 |  | GGATGGATGCACTACTGTCT | 51.8 | ACTACGAATTCGGACAGAGA | 49.7 |
| 42 | Nksrssr10_22560 |  | 0.80 | 22.56 | 6 |  | TTAAACTTCCAACTTTCCCA | 45.6 | AGGCTGTGTTTAGTTCCTGA | 49.7 |
| 43 | Nksrssr11_7483 | 11 | 0.83 | 7.483 | 6 | 4 | TCTCTCCTCTTGTTTGGCTC | 51.8 | ACACACCAACACGACCACAC | 53.8 |
| 44 | Nksrssr11_21630 |  | 0.77 | 21.63 | 7 |  | TATGTGTCGTGCCGTGTAG | 51.1 | GAGATTTGGAGGGAGAGGT | 51.1 |
| 45 | Nksrssr11_26523 |  | 0.85 | 26.523 | 7 |  | ACGACACGACCACCATAA | 48 | ACACGAAGAAGAGCACGA | 48 |
| 46 | Nksrssr11_27776 |  | 0.82 | 27.776 | 7 |  | TATTAGAGAAAGGGCGAAGAA | 48.5 | AGGGTCAAGAGACACACAGA | 51.5 |
| 47 | Nksrssr12_1.1 | 12 | 0.38 | 0.0011 | 2 | 0 | TGGTACTGTAGCAGCATGAG | 51.8 | TAGCTACAGACAGATTCGCA | 49.7 |
| 48 | Nksrssr12_1.7 |  | 0.67 | 0.0017 | 4 |  | AAACAAGGAGGAGGAGAAAG | 49.7 | GTTGGAGATGTAGAGCTTGG | 51.8 |
| 49 | Nksrssr12_21961 |  | 0.59 | 21.961 | 3 |  | GGCTCAACCTAGTTCTTCCT | 51.8 | CCCTGATTAGCCATACTTTG | 49.7 |
| 50 | Nksrssr12_23927 |  | 0.69 | 23.927 | 4 |  | GATGATGCAGAATCTCTTCG | 49.7 | GCTTCAGCTTCACTTTCTTC | 49.7 |
| Av. |  |  | 0.67 |  | 6.28 | 2.58 |  |  |  |  |

PIC = Polymorphic Information Content, Avg = avaerage, # alleles = number alleles per marker,

MP : Number of most poplymorphic markers ( PIC ≥ 0.70, # of polymorphic alleles ≥ 6)

Sl. No. corresponds to HvSSR No., for example, Sl. No. 1 (Nksrssr01_20380) represents Hv SSR-1, Sl. No. 2 (Nksrssr01_20427) represents Hv SSR-2 and so on.

S
